# Molecular preservation of 1.88 Ga Gunflint organic microfossils as a function of temperature and mineralogy

**DOI:** 10.1038/ncomms11977

**Published:** 2016-06-17

**Authors:** Julien Alleon, Sylvain Bernard, Corentin Le Guillou, Johanna Marin-Carbonne, Sylvain Pont, Olivier Beyssac, Kevin D. McKeegan, François Robert

**Affiliations:** 1 Institut de Minéralogie, de Physique des Matériaux et de Cosmochimie (IMPMC), Sorbonne Universités - CNRS UMR 7590, Muséum National d’Histoire Naturelle, UPMC Univ Paris 06, IRD UMR 206, 61 rue Buffon, 75005 Paris, France; 2 Univ Lyon, UJM Saint Etienne, Laboratoire Magma et Volcans, UBP, CNRS, IRD, 23 rue Dr Paul Michelon, 42100 St Etienne, France; 3 Department of Earth, Planetary and Space Sciences, University of California–Los Angeles, 595 Charles Young Drive East, Los Angeles, California 90095-1567, USA

## Abstract

The significant degradation that fossilized biomolecules may experience during burial makes it challenging to assess the biogenicity of organic microstructures in ancient rocks. Here we investigate the molecular signatures of 1.88 Ga Gunflint organic microfossils as a function of their diagenetic history. Synchrotron-based XANES data collected *in situ* on individual microfossils, at the submicrometre scale, are compared with data collected on modern microorganisms. Despite diagenetic temperatures of ∼150–170 °C deduced from Raman data, the molecular signatures of some Gunflint organic microfossils have been exceptionally well preserved. Remarkably, amide groups derived from protein compounds can still be detected. We also demonstrate that an additional increase of diagenetic temperature of only 50 °C and the nanoscale association with carbonate minerals have significantly altered the molecular signatures of Gunflint organic microfossils from other localities. Altogether, the present study provides key insights for eventually decoding the earliest fossil record.

## Abstract (web summary)

Thermal diagenesis is generally seen as detrimental to the preservation of organic biosignatures. Using synchrotron-based XANES data, Alleon *et al.* find preservation of the molecular signatures of organic microfossils from the 1.88 Ga Gunflint cherts.

## Graphical abstract

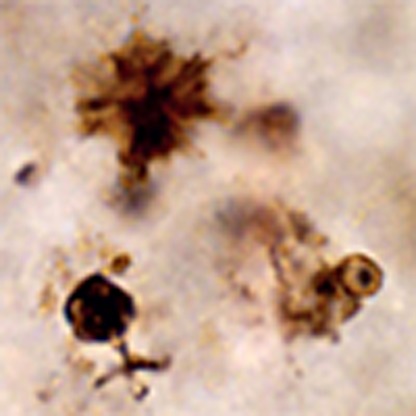

Although a number of studies have reported the morphological preservation of Precambrian organic microfossils, the search for the earliest traces of life on Earth has been and is still fraught with controversies^1^,^2^,^3^,^4^,^5^,^6^,^7^. Difficulties not only pertain to the inevitable degradation of biosignatures that occurred during the geological history of their host rocks^8^,^9^ but also to the possibility that abiotic chemical pathways yield organic microstructures exhibiting morphological and geochemical signatures very similar to biogenic ones^10^,^11^ as well as to the potential secondary/late contamination of the samples^12^,^13^.

Better understanding the impact of fossilization processes on biogenic molecular signatures is a major scientific challenge. Recent studies have investigated the influence of key parameters such as temperature and mineral matrix during experimental degradation of organics^14^,^15^,^16^,^17^. These studies have provided new information towards a generalized model of organic molecule degradation processes occurring during diagenesis and metamorphism. That said, few studies have relied on *in situ* measurements to discuss the molecular composition of Precambrian organic microfossils^18^,^19^,^20^,^21^.

The present study focuses on organic microfossils hosted in 1.88 Ga Gunflint cherts (that is, silica-rich rocks). The Gunflint Formation constitutes a cornerstone in life’s history: it shortly predates the earliest widely accepted evidence for fossil eukaryotes^22^,^23^,^24^. The Gunflint Formation is the middle unit of the Animikie group of Northwestern Ontario^25^,^26^,^27^. This 120-m-thick formation is conformably overlain by the Rove Formation and extends northeast–southwest for ∼175 km from Thunder Bay (Ontario, Canada) to northern Minnesota (USA)^25^,^26^,^27^.

The Gunflint cherts host what are considered among the best morphologically preserved Precambrian organic microfossils^4^,^5^,^6^. Although a high number of taxa have been reported (such as *Kakabekia* spp., *Eoastrion* spp. or *Eosphaera* spp. for instance), the dominant components of these microbiota are segmented tubular filaments (*Gunflintia* spp.) and coccoid-like microstructures (*Huroniospora* spp.)^27^,^28^,^29^,^30^. Although different levels of preservation have been recognized^29^, Gunflint organic microfossils usually serve as references for evaluating the biogenicity of older organic microstructures found in Archean rocks^4^,^5^,^6^.

The exceptional morphological preservation of Gunflint organic microfossils has been related to the very-low-grade metamorphism experienced by this formation^27^: burial temperatures of ∼150 °C have been estimated for the Gunflint Formation based on its mineral assemblage^25^ and microquartz oxygen isotopic composition^26^,^31^. Later on, hydrothermal circulation of oxygenated fluids resulting from the Penokean Orogeny (≈ 40 million years after the deposition of the Gunflint Formation^27^,^32^) and from the Duluth intrusion (emplaced during the 1.1-Ga Mesoproterozoic continental rifting^33^) may have locally increased temperatures^25^,^26^ without having induced any recrystallization of the microquartz matrix^34^,^35^. The 1.88-Ga Gunflint Formation thus constitutes a ‘natural laboratory’ very well suited to investigating the extent of the molecular preservation of organic microfossils submitted to different diagenetic and low-grade metamorphic conditions.

Here we report *in situ* structural and chemical investigations of organic microfossils from five Gunflint cherts that experienced different diagenetic temperatures. These five Gunflint cherts have been selected from the precambrian paleobiology research group (PPRG) collection^36^: Kakabeka Falls (PPRG 1,298), Schreiber Beach (PPRG 1,289), Triple Junction (PPRG 1,297), Mink Mountain (PPRG 1,374) and Discovery Point (PPRG 1,285).

Importantly, the present chemical investigations at the submicrometre scale have been performed on focused ion beam (FIB) ultrathin sections extracted from specimens found within freshly fractured fragments of samples that have never been embedded in epoxy, thereby avoiding contamination during sample preparation. Special caution has to be taken when adopting such sampling strategy, as it may confuse the precise recognition of organic microfossils versus blob-like detrital organics. As the organic microstructures investigated in the present study fulfill criteria for syngenicity as well as exhibit spectroscopic signatures thoroughly consistent with partially degraded biomolecules, these targets are hereinafter referred to as organic microfossils.

Optical microscopy has been performed on thin sections to document the presence of organic microfossils within the five Gunflint cherts investigated, whereas Raman microspectroscopy has been used to obtain information on the structure of their aromatic skeleton, to estimate the maximum temperature that they experienced^37^,^38^,^39^. Scanning and transmission electron microscopies (SEM and TEM) have been performed to document organic/mineral relationships. In parallel, synchrotron-based scanning transmission X-ray microscopy (STXM) coupled with X-ray absorption near edge structure (XANES) spectroscopy has been used to determine, at the submicrometre scale, the carbon and nitrogen speciation of Gunflint organic microfossils^40^,^41^. Based on the comparison with data collected on modern cyanobacteria (*Gloeobacter violaceus*) and modern micro-algae (*Euglena gracilis*), the present contribution shows that the molecular signatures of some organic microfossils from the 1.88-Ga Gunflint cherts have been exceptionally preserved (amide groups likely derived from protein compounds are detected), despite having experienced diagenetic temperatures of about 150–170 °C. The present study also demonstrates that slight increases of temperature and the mineral matrix can significantly alter the molecular signatures of biogenic remains.

## Results

### Chert mineralogy and microfossil morphology

**Figure 1:**
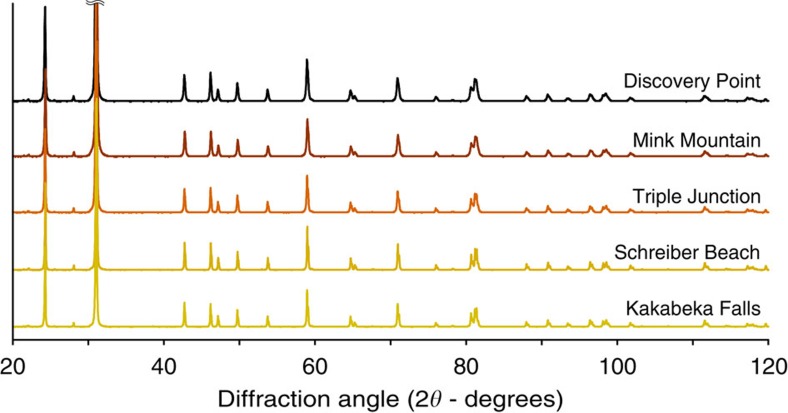
X-ray diffraction patterns of the five Gunflint cherts investigated. The five samples investigated display a similar mineralogical composition at the bulk scale (they are mainly composed of *α* quartz).

The Gunflint cherts investigated contain mainly microquartz as indicated by X-ray diffraction (quartz *α* with minor amount of quartz *β*; Fig. 1). The low concentration of minor phases (iron oxides and carbonates) prevents their detection. *In situ* secondary ion mass spectrometry measurements revealed that Schreiber Beach, Discovery Point and Mink Mountain microquartz have the same silicon isotopic composition (*δ*^30^Si=+1.75±0.35‰). This suggests a unique seawater silica source for Gunflint microquartz and confirms its pristine nature, that is, no hydrothermal silica is observed in the samples investigated^34^,^35^.

**Figure 2:**
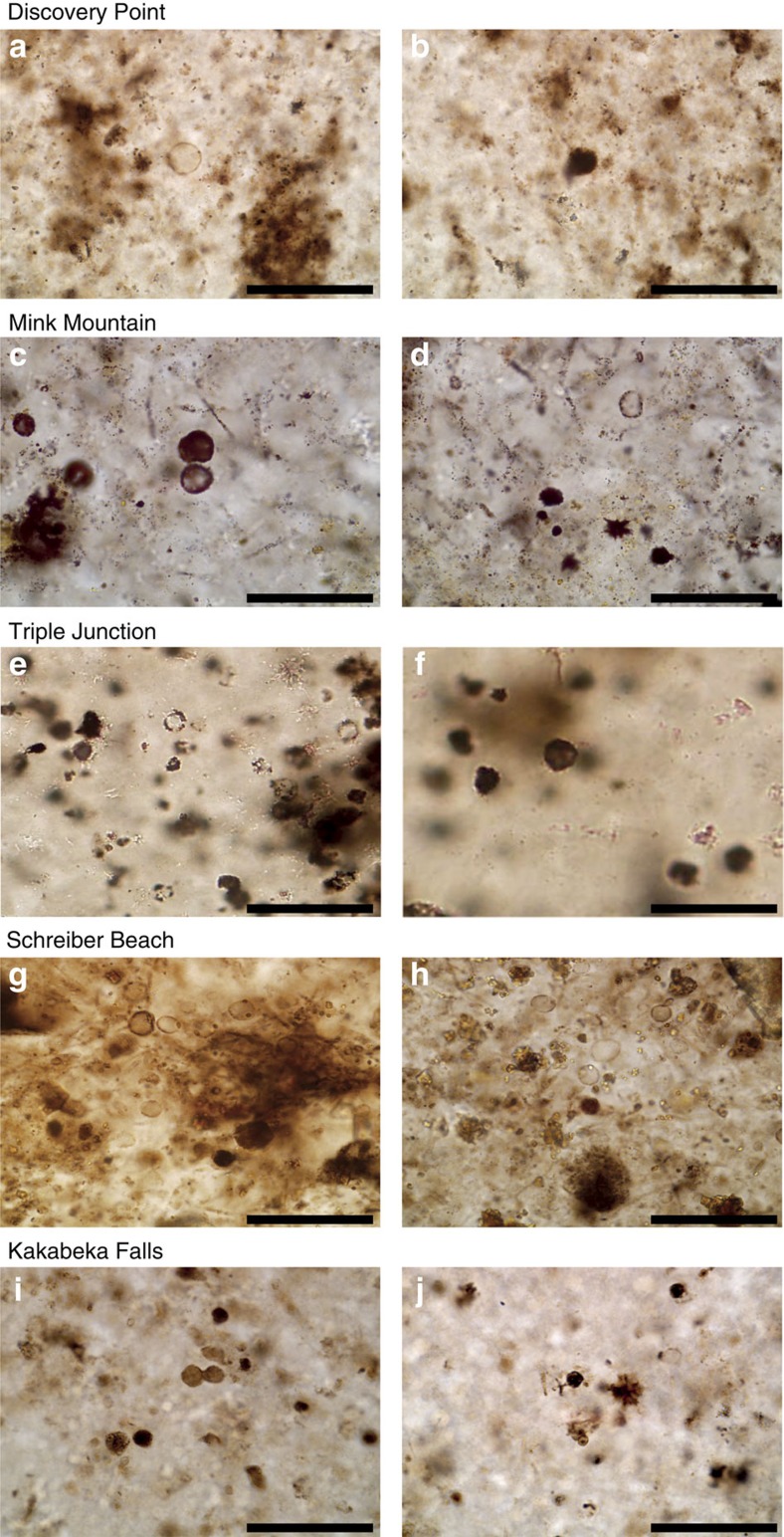
Photomicrographs of thin sections of the five Gunflint cherts investigated. These five samples contain spheroidal microfossils that appear more or less permineralized by silica and exhibit a more or less thick wall: (**a**,**b**) Discovery Point, (**c**,**d**) Mink Mountain, (**e**,**f**) Triple Junction, (**g**,**h**) Schreiber Beach, (**i**,**j**) Kakabeka Falls. Scale bars, 50 μm.

**Figure 3:**
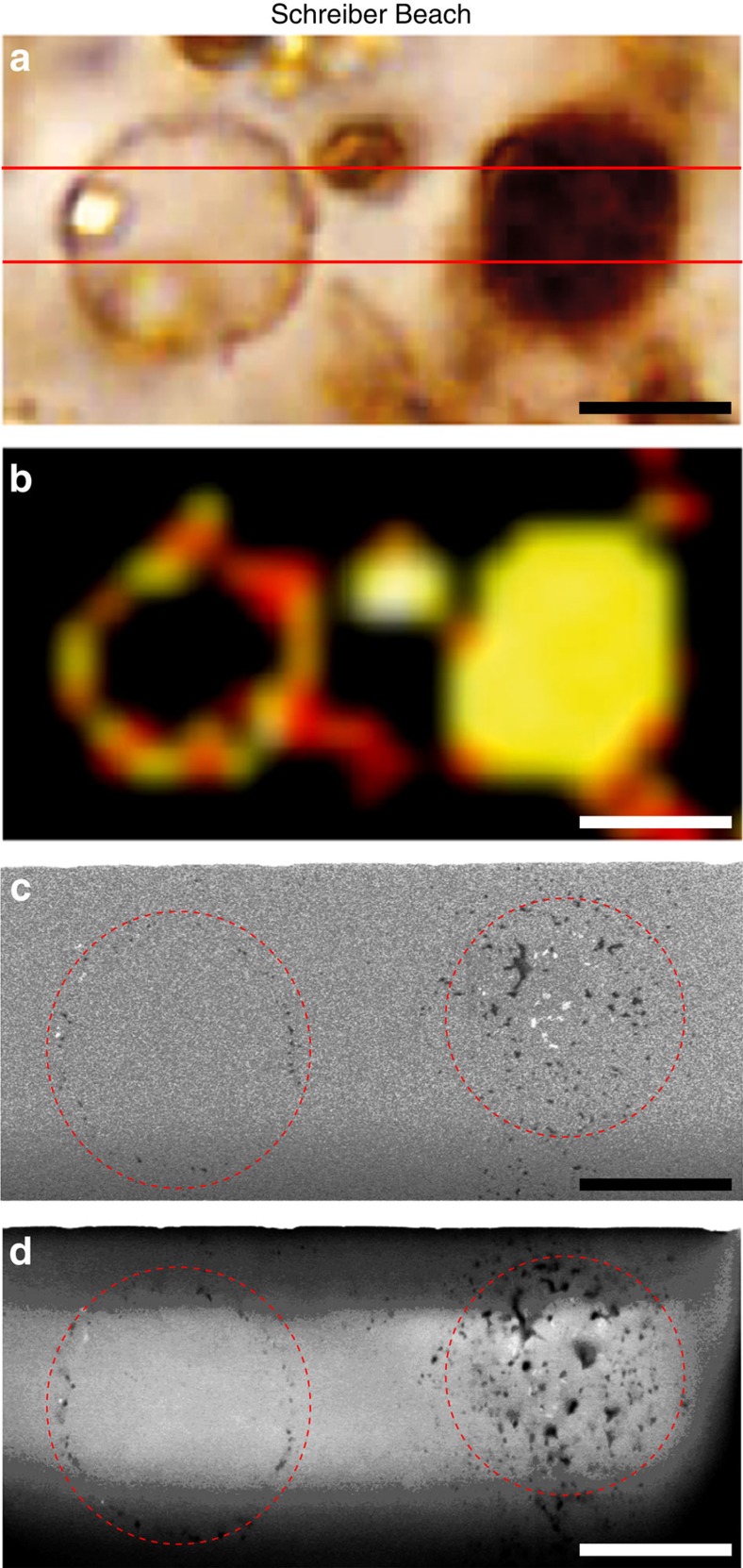
Raman mapping and FIB–SEM imaging of Schreiber Beach organic microfossils. (**a**) Photomicrograph of spheroidal organic microfossils from the Schreiber Beach chert and (**b**) corresponding Raman map showing the distribution of organic carbon. Red lines indicate the location of the cross-sections shown in **c**,**d**. Scale bars, 5 μm. (**c**,**d**) FIB–SEM images of cross-sections of the microfossils shown in **a**, illustrating that Gunflint spheroidal microfossils can be more or less permineralized by silica or filled by organics (which appear dark) and micrometric mineral phases (which appear bright). Scale bars, 5 μm.

The photomicrographs of thin sections of the Gunflint cherts investigated in the present study evidence the important diversity of morphologically preserved organic microfossils within these samples, together with the presence of organic masses with no particular morphology (Fig. 2). Consistently with previous observations^27^,^28^,^29^,^30^, the Gunflint microbiota appears dominated by more or less permineralized spheroidal microfossils of about 10–20 μm in diameter and exhibiting more or less thick walls (Fig. 2). Raman mapping experiments confirm the organic nature of these microfossils and FIB–SEM imaging evidence that although some spheroidal microfossils have been fully permineralized by silica, others can be filled by organics and micrometric mineral phases (Fig. 3).

### Microstructure of organic carbon

The Raman spectra collected on Schreiber Beach organic microfossils in the present study appear roughly similar to previous reports^1^,^3^. These spectra are typical of disordered carbons, with a composite G band (G+D2), a composite D band (D1+D4) and a D3 band in between^42^,^43^. Of note, there is no consensus on the exact physical or chemical significance of these so-called ‘defect’ bands^44^.

**Figure 4:**
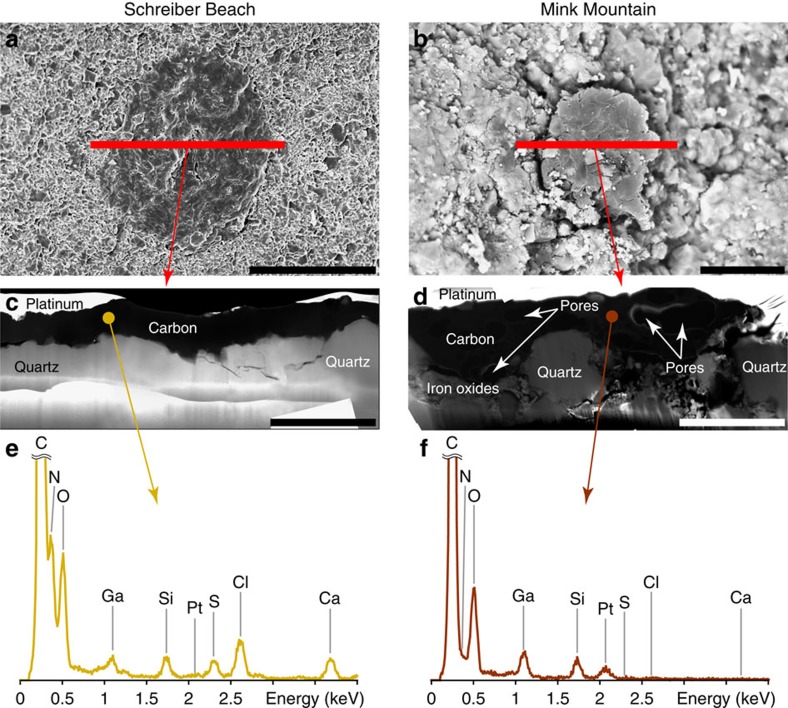
SEM and TEM analyses of Schreiber Beach and Mink Mountain organic microfossils. (**a**,**b**) SEM images of organic microfossils (dark). Red lines indicate where the FIB sections shown in **c** and **d** were extracted. Scale bars, 15 μm. (**c**,**d**) Scanning TEM (STEM) images of FIB sections extracted from the carbonaceous microfossils showing the textural relationships between the carbonaceous matter and the associated mineralogy. Organic carbon appears dark. Spots indicate where the EDXS data shown in **e** and **f** were collected. Scale bars, 5 μm. (**e**,**f**) TEM–EDX spectra acquired on the organic matter composing the microfossils. Schreiber Beach organic microfossils exhibit high nitrogen (N) and sulfur (S) contents compared to Mink Mountain organic microfossils.

All the Gunflint cherts investigated are quite homogeneous in terms of carbon structure, that is, in a given chert, all organic microfossils display a similar Raman spectrum (Fig. 4). Yet, slight differences can be observed from one chert to another (Fig. 4). In particular, the composite D band of Triple Junction, Mink Mountain and Discovery Point organic microfossils is more asymmetrical than the one of Kakabeka Falls and Schreiber Beach organic microfossils, probably due to a stronger contribution of the D1 band and a lower contribution of the D4 band (Fig. 4). Their composite G band is also slightly shifted, probably because of a stronger contribution of the G band and a lower contribution of the D2 band (Fig. 4). The Raman spectrum of the Discovery Point organic microfossils displays an additional peak at ∼1,090 cm^−1^ attributed to the presence of iron-rich calcium carbonates such as ankerites^45^,^46^.

The shape of the Raman signal collected on carbonaceous materials is directly related to their microstructure and has been shown to be related to the maximum temperature that they experienced^37^,^38^,^39^. The comparison of the Raman signatures of the Gunflint organic microfossils with a recent temperature calibration study^39^ indicates that Kakabeka Falls and Schreiber Beach have both experienced ∼150–170 °C during diagenesis, whereas Triple Junction, Mink Mountain and Discovery Point organic microfossils have been exposed to temperatures of ∼210–230 °C. The temperatures estimated here for the Kakabeka Falls and Schreiber Beach microfossils are in good agreement with previous estimations based the mineral assemblage^25^,^27^ and microquartz oxygen isotopic composition^26^,^31^ of Gunflint cherts.

### Submicrometric organic and mineral relationships

**Figure 5:**
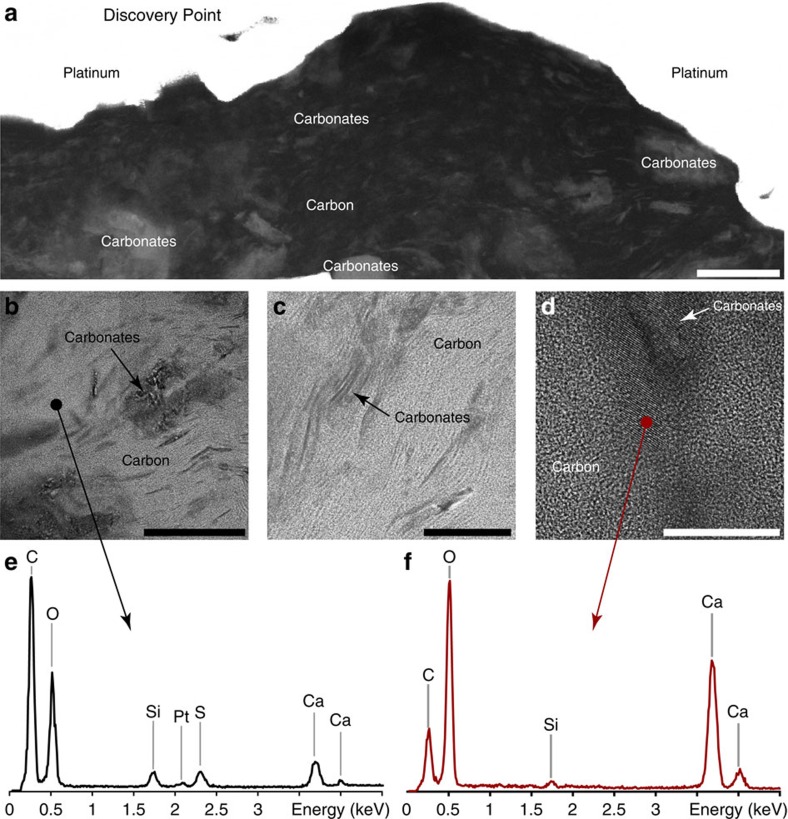
TEM analyses of Discovery Point organic microfossils. (**a**) Scanning TEM (STEM) image showing the nanoscale mixing of organic carbon and carbonates. Organic carbon appears dark. Scale bar, 250 nm. (**b**–**d**) High-resolution TEM images showing details of this nanoscale association. Spots indicate where the EDXS data were collected. Scale bars, 200 nm (**b**), 100 nm (**c**) and 20 nm (**d**). (**e**,**f**) TEM–EDX spectra acquired on the organic matter constituting the microfossils and on the associated calcium carbonates.

**Figure 6:**
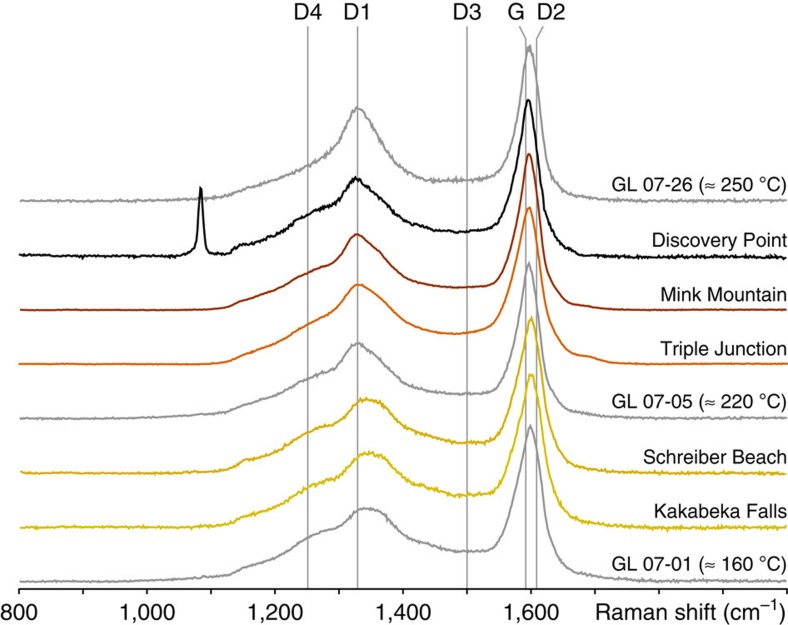
Representative Raman spectra of Gunflint organic microfossils. These spectra are typical of disordered carbons, with a composite G band (G+D2), a composite D band (D1+D4) and a D3 band in between. Grey spectra are data from the calibration series published by Lahfid *et al.*^39^. The peak at ∼1,090 cm^−1^ in the Raman spectrum of Discovery Point organic microfossils is attributed to iron-rich calcium carbonates such as ankerites^45^,^46^.

FIB ultrathin sections have been extracted from the walls of organic microfossils found using SEM within freshly fractured fragments of cherts for further characterization using TEM and STXM. Again, special caution has been taken for such sampling strategy, as it may confuse the precise recognition of organic microfossils versus blob-like detrital organics. TEM observations highlight textural differences among the organic microfossils investigated: although the Schreiber Beach organic microfossils appear almost free of porosity at the nanoscale, the Mink Mountain organic microfossils exhibit nanoporosity (Fig. 5). TEM–energy dispersive X-ray spectrometry (EDXS) measurements show that Schreiber Beach organic microfossils contain a significantly higher amount of nitrogen and sulfur than those from Mink Mountain (Fig. 5). In addition, TEM observations confirm the presence of iron oxides around Mink Mountain organic microfossils (Fig. 5), in agreement with previous observations^27^, and of calcium carbonates within Discovery Point organic microfossils (Fig. 6). Although iron oxides possibly precipitated during postdepositional circulation of oxygenated fluids^27^, carbonates may have precipitated either before or as a result of the degradation of organics.

### Nitrogen-to-carbon atomic ratio

**Figure 7:**
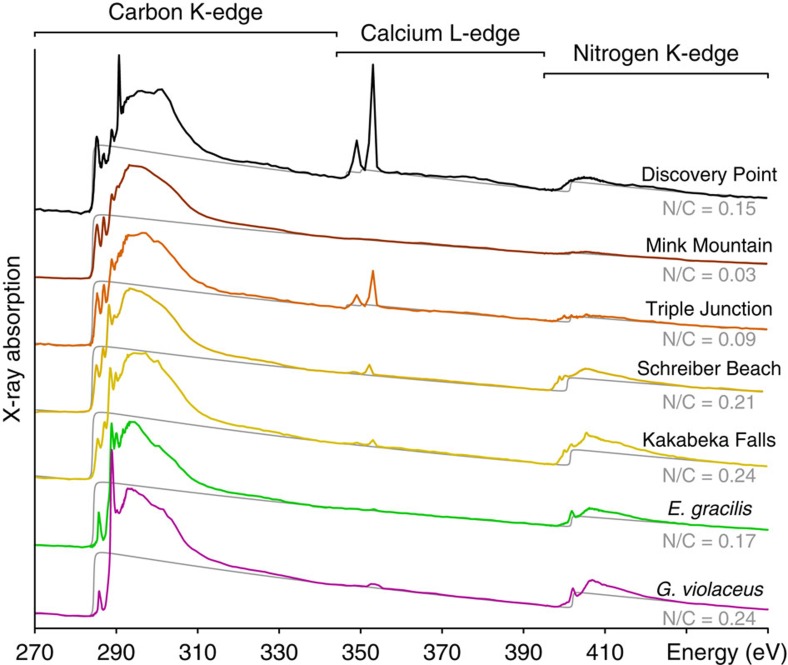
X-ray absorption spectra of Gunflint organic microfossils. The atomic N/C ratios (±0.010) shown here have been estimated following the methodology developed by Alleon *et al.*^47^. The absorption spectra of modern cyanobacteria (*G. violaceus*) and of modern micro-algae (*E. gracilis*) are shown for comparison. Absorption features observed in the 340–360 eV range indicate the presence of calcium.

The nitrogen-to-carbon atomic ratio (N/C) of the Gunflint organic microfossils investigated have been estimated from their X-ray absorption spectra^47^. Organic microfossils from Kakabeka Falls and Schreiber Beach have a higher N/C (0.24 and 0.21±0.01, respectively) than the ones from Triple Junction and Mink Mountain (0.09 (0.05 after correction, see below) and 0.03±0.01, respectively) (Fig. 7). Despite more significant molecular degradation (see below), Discovery Point organic microfossils exhibit a quite high N/C (0.15±0.01). For comparison, a value of 0.17±0.01 is obtained from the X-ray absorption spectrum of modern micro-algae, whereas a value of 0.24±0.01 is estimated for modern cyanobacteria (Fig. 7).

The absorption signal at the calcium *L*-edge (340–360 eV) shows that all the Gunflint organic microfossils, except the ones from Mink Mountain, contain variable amounts of calcium. In addition, Kakabeka Falls and Triple Junction organic microfossils appear associated with calcium and/or potassium nitrates (Ca(NO_3_)_2_ and KNO_3_) as indicated by their nitrogen speciation (see below). If all the calcium in the Triple Junction organic microfossils occurred as calcium nitrates, the inorganic nitrogen contribution corresponding to nitrates could be directly derived from the calcium content estimated from the intensity of the absorption at the calcium *L*-edge. Subtracting this inorganic nitrogen quantity from the total nitrogen contribution would lead to an organic N/C of≈0.05±0.01 for Triple Junction organic microfossils, that is, to a value comparable to the N/C of Mink Mountain organic microfossils. As the calcium and potassium contents of Kakabeka Falls organic microfossils are very low, such correction would not significantly modify the estimated organic N/C.

### Carbon speciation and molecular signatures

**Figure 8:**
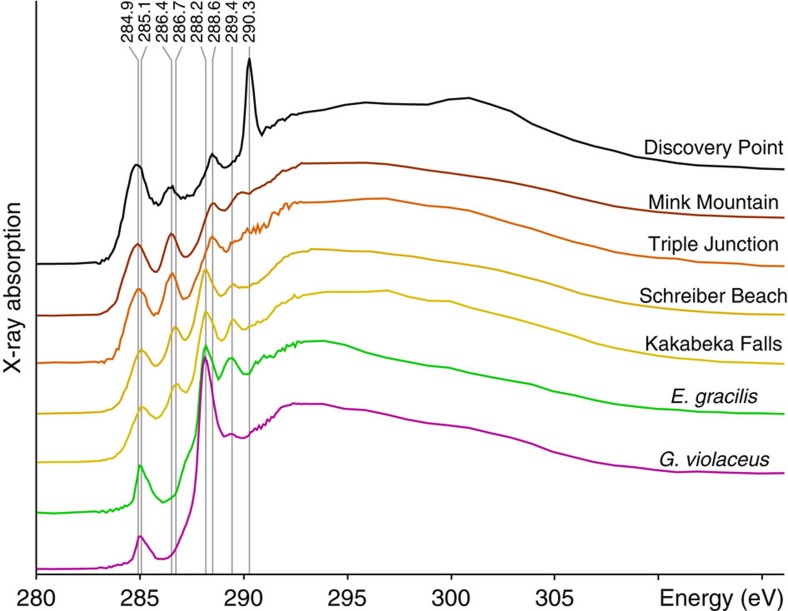
C-XANES spectra of Gunflint organic microfossils. The C-XANES spectra of modern cyanobacteria (*G. violaceus*) and of modern micro-algae (*E. gracilis*) are shown for comparison. All spectra are normalized to the total carbon quantity. Absorption features at 285.1, 286.4, 286.7, 288.2, 288.6, 289.4 and 290.3 eV are attributed to electronic transitions of aromatic and/or olefinic carbons, carbons bonded with heteroatoms, carbonyl or phenolic groups, amide groups, carboxylic groups, hydroxylated carbons and carbonate groups, respectively.

After normalization to the total carbon quantity, Kakabeka Falls and Schreiber Beach organic microfossils exhibit C-XANES spectra sharing similar features with spectra of modern cyanobacteria and modern micro-algae (Fig. 8), that is, an intense peak at 288.2 eV attributed to the presence of amide functional groups, a second peak at 289.4 eV attributed to the presence of hydroxyl functional groups and a third one, less intense, centred at 285.1 eV and attributed to the presence of aromatic and/or olefinic functional groups^15^,^48^. Compared with modern microorganisms, Kakabeka Falls and Schreiber Beach organic microfossils display an additional absorption feature in their C-XANES spectra centred at 286.7 eV and likely to be attributed to the presence of carbonyl or phenolic functional groups^49^,^50^.

The C-XANES spectra of Triple Junction and Mink Mountain organic microfossils are quite different (Fig. 8). The absorption feature attributed to the presence of aromatic and/or olefinic groups is broader, more intense and shifted to 284.9 eV, probably due to a higher content of olefins^51^ and/or of heteroaromatic moieties such as benzoquinones^48^. The absorption peaks at 286.7, 288.2 and 289.5 eV do not contribute as significantly to the signal. Instead, two other features are observed: a peak centred at 286.4 eV, attributed to the presence of unsaturated bond between carbon and heteroatoms such as nitrogen, sulfur and/or oxygen, and a peak centred at 288.6 eV attributed to the presence of carboxylic functional groups^49^,^50^.

The C-XANES spectrum of Discovery Point organic microfossils exhibits absorption peaks at the same energies as those of Triple Junction and Mink Mountain (Fig. 8). Yet, the aromatic absorption feature (centred at 284.9 eV) is broader and more intense, and an additional sharp and intense peak at 290.3 eV indicates the nanoscale association between organics and carbonates^45^,^52^,^53^, probably calcium carbonates as indicated by EDXS data (Fig. 6).

### Nitrogen speciation and molecular signatures

**Figure 9:**
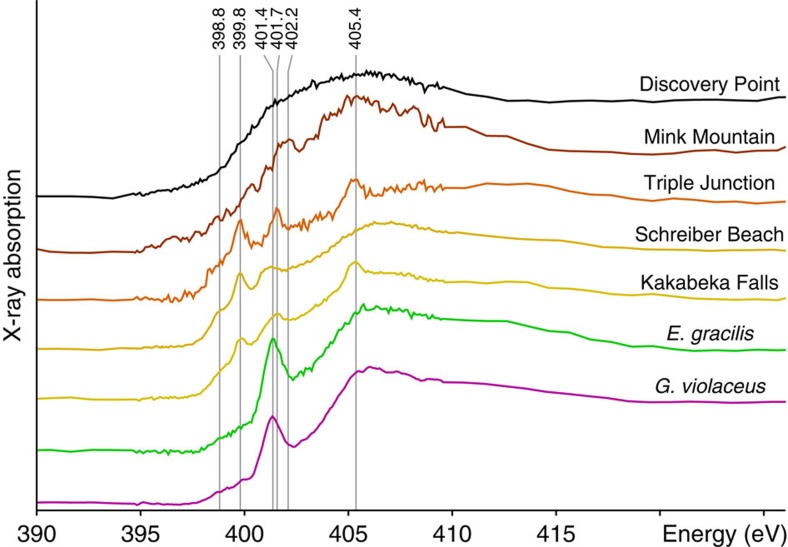
N-XANES spectra of Gunflint organic microfossils. The N-XANES spectra of modern cyanobacteria (*G. violaceus*) and of modern micro-algae (*E. gracilis*) are shown for comparison. All spectra are normalized to the total nitrogen quantity. Absorption features at 398.8, 399.8, 401.4 and 402.2 eV are attributed to the presence of aromatic nitrogen, amide, imine and nitrile groups. Absorption peaks at 401.7 and 405.4 eV are attributed to inorganic nitrogen contained in nitrates.

Similar to C-XANES data, the N-XANES spectra have been normalized to the total nitrogen quantity to facilitate their comparison. Kakabeka Falls, Schreiber Beach and Triple Junction organic microfossils exhibit a broad absorption feature at 401.4 eV, possibly indicating the presence of amide, imine and nitrile groups, as well as a well-defined peak at 399.8 eV and a shoulder around 398.8 eV, which may denote the presence of a significant amount of nitrogen within aromatic moieties^54^,^55^ (Fig. 9). Similar absorption features can be observed in the N-XANES spectrum of modern cyanobacteria and modern micro-algae, and are usually attributed to the presence of amide (peak at 401.4 eV), imine, nitrile and/or aromatic nitrogen^54^ (Fig. 9). Two additional absorption features are observed at 401.7 and 405.4 eV in the N-XANES spectra of Kakabeka Falls and Triple Junction organic microfossils (Fig. 9). These peaks can be attributed to the presence of calcium and/or potassium nitrates^54^. These microfossils thus contain both organic and inorganic nitrogen. The only absorption peak that can be observed in the N-XANES spectrum of Mink Mountain organic microfossils occurs at 402.2 eV and may be attributed to the presence of pyrrole^54^,^55^ (Fig. 9). The lower signal-to-noise ratio is due to the lower nitrogen content of these microfossils. In contrast, despite a high N/C ratio, the N-XANES spectrum of Discovery Point organic microfossils does not display any absorption peak (Fig. 9).

## Discussion

The 1.88-Ga Gunflint cherts belong to the most famous Precambrian organic microfossil-rich formation^4^,^5^,^6^. Previous studies based on TEM-based electron energy loss spectroscopy (EELS) experiments have not reported the presence of nitrogen-bearing functional groups within Gunflint organic microfossils^2^,^3^,^18^, probably because of the low spectral resolution of this technique and potentially because of electron radiation damage (the radiation dose received by organics has been shown to be typically 100–1,000 times higher in TEM-based EELS than in STXM-based XANES spectroscopy^56^). The rare STXM-based C-XANES data reported in the literature on Gunflint samples have not been collected *in situ* but on powdered samples^2^,^3^, which prevented the demonstration of the syngenicity of the measured organics.

Taking advantage of the unique capabilities of STXM-based XANES spectroscopy at the carbon and nitrogen K edges to perform *in situ* experiments at the submicrometre scale, the present study shows that, in addition to the fine-scale morphologies, the molecular biosignatures of some Gunflint organic microfossils have been exceptionally preserved. In fact, despite the 1.88-Gyr-long geological history that they experienced, Kakabeka Falls and Schreiber Beach organic microfossils exhibit C- and N-XANES spectra sharing strong similarities to those of modern cyanobacteria and modern micro-algae. Despite a higher content of aromatic compounds compared to modern microorganisms, these microfossils exhibit a quite high content of oxygen-based functional groups (carbonyl, phenolic, carboxylic and hydroxyl groups). In addition, these microfossils still contain amide functional groups (absorption feature at 288.2 eV), which were likely to be involved in the proteinaceous compounds synthetized by the once living organisms^15^,^52^,^53^.

Kakabeka Falls and Schreiber Beach organic microfossils exhibit quite high N/C values. These values may be secondary, that is, may result from diagenetic processes. In fact, inorganic nitrogen in fluids can be incorporated within kerogen molecular structures during diagenesis at temperatures as low as 100 °C^57^. Yet, although higher than those of modern micro-algae, the N/C of Kakabeka Falls and Schreiber Beach organic microfossils are comparable to those of modern cyanobacteria. It thus can be assumed that the Gunflint organic microfossils initially exhibited high N/C values as do modern cyanobacteria and some modern marine microorganisms showing N/C as high as 0.25–0.30 (refs 58, 59). The high N/C of Kakabeka Falls and Schreiber Beach organic microfossils may thus result from their exceptional preservation.

The molecular signatures of Triple Junction, Mink Mountain and Discovery Point organic microfossils have not been that well preserved. In fact, the Raman and XANES data reported here show that these organic microfossils are more ‘mature’ than the ones from Kakabeka Falls and Schreiber Beach, that is, they exhibit a higher aromaticity and contain less sulfur-, nitrogen- and oxygen-rich moieties^49^,^50^. In particular, these organic microfossils do not seem to contain amide, hydroxyl nor carbonyl functional groups.

One explanation could be that the organic microfossils investigated here were initially chemically different and/or experienced variable decay^60^. In other words, they may have been originally composed of different organics, which may have followed different reaction pathways during diagenesis. Yet, morphologies, bulk mineralogy and silicon isotopic compositions suggest comparable depositional and burial histories.

Another possibility would be that they have experienced different oxidation conditions: oxic conditions have been shown to be detrimental to the preservation of biosignatures^14^ and the association of Kakabeka Falls, Triple Junction and Mink Mountain organic microfossils with nitrates and iron oxides suggests that oxygenated fluids have circulated^27^. Yet, Schreiber Beach and Kakabeka Falls organic microfossils exhibit very similar molecular signatures, even though only the latter are associated with nitrates. Furthermore, even though nitrates are associated to Kakabeka Falls and Triple Junction organic microfossils, their molecular signatures are very different. The circulation of oxygenated fluids thus cannot be seen as the main process having an impact on the molecular degradation of the investigated Gunflint organic microfossils.

As the differences reported here are very similar to those resulting from thermal maturation^49^,^50^, the simplest explanation remains that they result from the different burial temperature conditions experienced by the organic microfossils investigated. Interestingly, with increasing maturity, a shift from 285.1 to 284.9 eV is observed for the aromatic/olefinic peak whereas the phenolic/carbonyl peak shifts from 286.7 to 286.4 eV. These shifts may result from the incorporation of heteroatoms in newly condensed aromatic units and from condensation reactions between amino acids and phenolic groups, respectively^49^,^50^. In any case, the present contribution demonstrates that a slight increase of diagenetic temperatures may be responsible for the significant degradation of fossilized molecular signatures.

Regardless of their high N/C, to which inorganic nitrogen may contribute, the molecular signature of Discovery Point organic microfossils appears less pristine, that is, more degraded, than those of Triple Junction and Mink Mountain organic microfossils (Figs 8 and 9), even though they experienced similar diagenetic temperatures (that is, 210–230 °C; Fig. 4). The possibility that Discovery Point organic microfossils were initially different from the other investigated Gunflint cherts cannot be entirely ruled out, even though nothing supports it (see above). In contrast, as illustrated by the similar XANES signatures of modern micro-algae (*E. gracilis*) and modern cyanobacteria (*G. violaceus*), prokaryotes such as the ones fossilized within the investigated Gunflint cherts probably originally exhibited very similar XANES signatures.

The presence of nitrates might explain the better preservation of Triple Junction organic microfossils compared with those of Discovery Point. Yet, Kakabeka Falls and Triple Junction organic microfossils are also associated with nitrates but exhibit C-XANES spectra very similar to the nitrate-free Schreiber Beach and Mink Mountain organic microfossils, respectively (Figs 8 and 9). The postdepositional circulation of oxygenated fluids, which led to the precipitation of iron oxides^27^, may have also been responsible for the molecular degradation of organic microfossils. Yet, Triple Junction and Mink Mountain organic microfossils exhibit very similar C-XANES spectra (Fig. 6), even though only the latter are associated with iron oxides. Thus, the degree of molecular preservation of Gunflint organic microfossils is not correlated to the presence of nitrates and iron oxides.

Alternatively and more probably, the higher maturity of Discovery Point organic microfossils could be related to the nanoscale association between organics and carbonates revealed by TEM, Raman and XANES analyses (Figs 3, 4 and 6). Among the Gunflint organic microfossils investigated, only the ones from Discovery Point exhibit such nanoscale association. It has previously been reported that the presence of calcium carbonates during high-temperature organic maturation processes may favour the formation of turbostratic (graphitic) carbons^37^,^61^, through the formation of calcium hydroxide and calcium carbide, especially under nitrogen-rich atmosphere^62^,^63^. This reaction pathway has been observed at high temperature; thus, such a scenario remains highly speculative in the present case. In any case, although dedicated and thorough experimental investigations appear required, the present study illustrates the potential impact of mineral phases on the preservation/degradation of fossilized molecular signatures.

Altogether, the present contribution shows that the molecular signatures of the organic microfossils from the 1.88-Ga Gunflint cherts have been preserved, although they experienced temperatures of about 150–170 °C. Such preservation can be qualified as exceptional, as amide groups derived from protein compounds can still be detected. Amide groups are indeed generally lost during the very first stages of burial, either consumed by heterotroph organisms or thermally degraded at low temperature (<< 100 °C)^64^. Although a number of taphonomic processes may be invoked, it can be assumed that this exceptional molecular preservation result from the early silicification of Gunflint microbiota. Indeed, even though Kakabeka Falls and Schreiber Beach organic microfossils have experienced diagenetic temperatures similar to overmature clay-rich gas shales, their molecular signatures are significantly better preserved than those corresponding to overmature kerogens^65^. The present study thus illustrates that Precambrian cherts having experienced relatively low-grade metamorphism may contain (at least partially) chemically preserved remains of ancient life. As slight increases of temperature can strongly modify the molecular signatures of biogenic remains, documenting the carbon and nitrogen speciation of organic microfossils and estimating the temperature that they experienced should be done in parallel. We believe that the analytical strategy adopted here, combining Raman and STXM-based XANES spectroscopies, is an illustration of the technical and conceptual advances of Precambrian palaeontology that will eventually lead to the elucidation of a large part of the mysteries that still shroud the early fossil record.

## Methods

### X-ray diffraction

The bulk mineralogical composition of the Gunflint cherts investigated has been determined using the X-ray diffractometer (Panalytical X'pert Pro) operating at IMPMC (Paris, France) with 40 kV and 40 mA Co Kα radiation. Sample analyses have been carried out on finely ground powders deposited on a silicon sample holder, in the 20–120° 2*θ* angle range, with a step size of 0.016° (2*θ*) for a total counting time per sample of about 6 h. X-ray diffraction patterns have been analysed using the Eva software (Bruker) for background subtraction and peak finding.

### Secondary ion mass spectrometry

Silicon isotopic compositions (*δ*^30^Si) of microquartz have been classically measured on the Cameca 1270 ion microprobe operating at UCLA (CA, USA). The measured *δ*^30^Si values have been corrected using in house quartz standards (QZCRWU and chert Miocene) and are reported here as per mil deviations from the international standard NBS28. Samples have been sputtered with a 25 μm size Cs^+^ primary beam of 30 nA intensity using a 10-kV acceleration voltage. The mass resolving power was set at *M*/Δ*M*∼4,000. Faraday cups have been used to measure simultaneously ^28^Si^−^ and ^30^Si^−^ ions. Field diaphragm and magnetic field have been automatically centred during the analyses. A pre-sputtering of 60 s and 40 × 5-s-long cycles of acquisition have led to a counting statistic of <0.1‰ and an external reproducibility of ∼0.3‰ (1*σ*).

### Scanning electron microscopy

SEM has been used to locate the organic microstructures within the silica matrix of the investigated Gunflint cherts for subsequent *in situ* extraction using FIB milling. To minimize contamination that may come from sample preparation, freshly fractured fragments of samples have been directly observed after having been mounted on aluminum stubs without any additional preparation, except gold coating. SEM observations have been performed on an SEM–field emission gun ultra 55 Zeiss (IMPMC, Paris, France) at a 15-kV accelerating voltage and a working distance of 7.5 mm. SEM images have been collected with both secondary electron (SE2) and angle-selective backscattered detectors. Elemental maps have been collected using EDXS.

### Raman microspectroscopy and Raman mapping

Raman data have been obtained with a Renishaw INVIA microspectrometer (IMPMC, Paris, France). Raman microspectroscopy measurements have been directly performed on freshly fractured samples at constant room temperature using the 514.5-nm wavelength of a 50-mW Modulaser Argon laser (green laser) focused on the sample through a Leica DM LM microscope with a long working distance × 100 objective (numerical aperture=0.75). This configuration yields a horizontal resolution of ≈1 μm for a laser power delivered at the sample surface always below 1 mW to prevent irreversible laser-induced thermal damage^43^. A circularly polarized laser using a quarter wavelength plate allows limiting polarization effects. Light is dispersed by a grating with 1,800 lines per mm and the signal is analysed with a RENCAM CCD (charge-coupled device) detector. Ten to fifteen spectra have been collected for each sample. Dynamic Raman hyperspectral mapping has been performed using an equivalent spectrometer relying on the synchronization of CCD measurements with *x*,*y* motorized stage displacements^66^. At each point, a correlation index between the measured spectrum and a reference spectrum of organics is calculated. Points displaying high indexes are assigned a colour, from red to yellow/white.

### FIB milling and FIB-SEM imaging

FIB ultrathin sections have been extracted from the walls of organic microfossils using an FEI Strata DB 235 (IEMN, Lille, France). This extraction procedure maintains textural integrity, even in the case of loosely consolidated materials, and prevents shrinkage and deformation of microscale to nanoscale pores, even in the case of highly sensitive materials^67^. Milling at low Ga-ion currents has allowed preventing common artefacts such as local gallium implantation, mixing of components, creation of vacancies or interstitials, creation of amorphous layers, local composition changes or redeposition of the sputtered material on the sample surface and significant changes in the speciation of complex carbon-based polymers^40^,^68^. FIB-SEM imaging (backscattered electrons) of FIB milled trenches have been performed using a Zeiss Crossbeam Auriga FIB-SEM (IPGP, Paris, France).

### Transmission electron microscopy

TEM analyses have been performed on FIB sections to document the textural nature of the investigated Gunflint organic microfossils and identify the mineral phases with which organics are closely associated at the nanoscale. TEM observations have been performed with a JEOL 2100 field emission gun microscope (IMPMC, Paris, France) operating at 200 kV. Scanning TEM Z-contrast imaging has been performed using the high-angle annular dark field mode. High-resolution TEM images have been collected using the bright-field mode, allowing us to resolve the crystalline planes (of the order of 0.1 nm) of the different phases.

### X-ray absorption spectroscopy

XANES data have been collected on the STXM 10ID-1 beamline (SM beamline)^69^ at the Canadian Light Source. The 10ID-1 beamline works in the soft X-ray energy range (130–2,500 eV) and is based on an elliptically polarized undulator. The Canadian Light Source storage ring is operated at 2.9 GeV and between 250 and 150 mA current. The microscope chamber is first pumped down to 100 mTorr after sample insertion and back-filled with He gas. A 100-nm-thick titanium filter is used to remove the contribution of second-order light. Energy calibration is done using the well-resolved 3p Rydberg peak of gaseous CO_2_ at 294.96 eV for the C K-edge and using the 1 s→π* photoabsorption resonance of gaseous N_2_ at 400.8 eV for the N K-edge.

X-ray absorption spectroscopy has been performed by collecting image stacks with a spatial resolution of 15 nm, that is, by rastering selected areas of samples in the x–y directions at energy increments of 1 eV over the 270–450 eV energy range using the low-energy grating of the 10ID-1 SM beamline. Additional image stacks have been collected at energy increments of 0.1 eV over the carbon (270–340 eV) and the nitrogen (390–450 eV) absorption ranges, to resolve the fine structures near the C and N K-edges (XANES spectroscopy). Stack measurements have been performed with a dwell time of ≤1 ms per pixel to prevent irradiation damage. Alignment of images of stacks and extraction of XANES spectra have been done using the aXis2000 software (ver2.1n). Spectral peak positions, intensities and widths have been determined using the Athena software package^70^. The C- and N-XANES spectra shown in the present contribution correspond to homogeneous organic-rich areas of several hundreds of square nanometres.

### Data availability

The data that support the findings of this study are available from the corresponding author (S.B.) upon request.

## Additional information

**How to cite this article:** Alleon, J. *et al.* Molecular preservation of 1.88 Ga Gunflint organic microfossils as a function of temperature and mineralogy. *Nat. Commun.* 7:11977 doi: 10.1038/ncomms11977 (2016).
