# Erratum: Molecular preservation of 1.88 Ga Gunflint organic microfossils as a function of temperature and mineralogy

**DOI:** 10.1038/ncomms16147

**Published:** 2017-08-14

**Authors:** Julien Alleon, Sylvain Bernard, Corentin Le Guillou, Johanna Marin-Carbonne, Sylvain Pont, Olivier Beyssac, Kevin D. McKeegan, François Robert

Nature Communications 7 Article number: 11977 ; DOI: 10.1038/ncomms11977 (2016); Published 06 17 2016; Updated 08 14 2017

This Article contains errors in the figure numbering and referencing that were introduced during the production process. Figures 4, 5 and 6 should be labelled as Figs 5, 6 and 4, respectively. In paragraph 9 of the Discussion section, the penultimate sentence should read ‘Yet, Triple Junction and Mink Mountain organic microfossils exhibit very similar C-XANES spectra (Fig. 8), even though only the latter are associated with iron oxides.’—referring to ‘Fig. 8’ not ‘Fig. 6’. In paragraph 10 of the Discussion section, the first sentence should read ‘Alternatively and more probably, the higher maturity of Discovery Point organic microfossils could be related to the nanoscale association between organics and carbonates revealed by TEM, Raman and XANES analyses (Figs 4,6 and 8).’—referring to ‘Figs 4,6 and 8’ and not ‘Figs 3,4 and 6’.

